# Advancements in the characterisation of oligonucleotides by high performance liquid chromatography‐mass spectrometry in 2021: A short review

**DOI:** 10.1002/ansa.202100066

**Published:** 2022-04-10

**Authors:** Fabien Hannauer, Rachelle Black, Andrew D. Ray, Eugen Stulz, G. John Langley, Stephen W. Holman

**Affiliations:** ^1^ Department of Chemistry, Faculty of Engineering and Physical Sciences University of Southampton Southampton UK; ^2^ New Modalities Product Development Pharmaceutical Technology & Development, Operations, AstraZeneca Macclesfield UK; ^3^ Chemical Development Pharmaceutical Technology & Development, Operations, AstraZeneca Macclesfield UK

## Abstract

The first oligonucleotide therapeutic was approved by the Food and Drug Administration in 1998, and since then, 12 nucleic acids have been commercialised as medicines. To be approved, the oligonucleotides need to be identified and characterised as well as its related impurities. Different methods exist, but the most commonly used is ion‐pairing reversed‐phase liquid chromatography with tandem mass spectrometry. The separation obtained depends on the mobile phase and column used. Other methods have been developed, notably by using hydrophilic interaction chromatography and two‐dimensional high performance liquid chromatography. Furthermore, ion‐pairing reversed‐phase high performance liquid chromatography ultra‐violet spectroscopy detection and mass spectrometry has been optimised for the analysis of methylated nucleobases due to the utilisation of this modification in the drugs. This review covers the recent advancements in the analysis and characterisation of oligonucleotides in 2021 by high performance liquid chromatography mass spectrometry, notably by hydrophilic interaction chromatography and two‐dimensional liquid chromatography but also the different parameters that influence the analysis by ion‐pairing reversed‐phase high performance liquid chromatography, the characterisation of methylated nucleobases, and the recent software developed for oligonucleotides.

**Abbreviations**
^1^D: first dimension
^2^D: second dimension
2D‐LC: two‐dimensional‐liquid chromatography
7PrG: 7‐propyl guanine
7BuG: 7‐butyl guanine
6mA: *N*6‐methyladenine,
4mC: *N*4‐methylcytosine
5mC: C5‐methylcytosine
AA: ammonium acetate
ABC: ammonium bicarbonate
AF: ammonium formate
AX: anion‐exchange
CID: collision‐induced dissociation
ESI: electrospray ionisation
FA: formic acid
FDA: Food and Drug Administration
FLP: full‐length product
HCD: high‐energy C‐trap dissociation
HFIP: hexafluoropropan‐2‐ol
HILIC: hydrophilic interaction chromatography
IMS: ion mobility spectrometry
IP‐RPLC: ion‐pairing‐reversed‐phase liquid chromatography
IPR: ion‐pairing reagent
LNA: locked nucleic acids
MHC: multiple‐heart‐cutting
MOE: 2′‐*O*‐methoxyethyl
MRM: multiple reaction monitoring
MS: mass spectrometry
%NCE: % normalized collisional energy
NFTB: nonafluoro‐*tert* *‐butyl alcohol*
OA: octylamine
PA: propylamine
PRM: parallel reaction monitoring
QN: quinine
siRNA: small interfering RNA
TEA: triethylammonium
TEAA: triethylammonium acetate
TEAB: tetraethylammonium bromide
TEAP: triethylammonium phosphate
TPAA: tripropylammonium acetate
tRNA: transfer RNA
tSIM: targeted selected ion monitoring

## 1 INTRODUCTION

**FIGURE 1:**
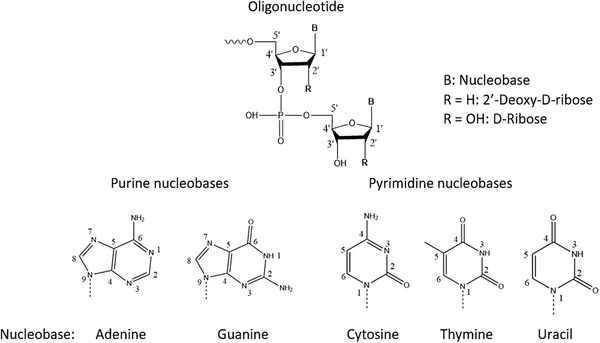
Structures of nucleobases and oligonucleotide

**FIGURE 2:**
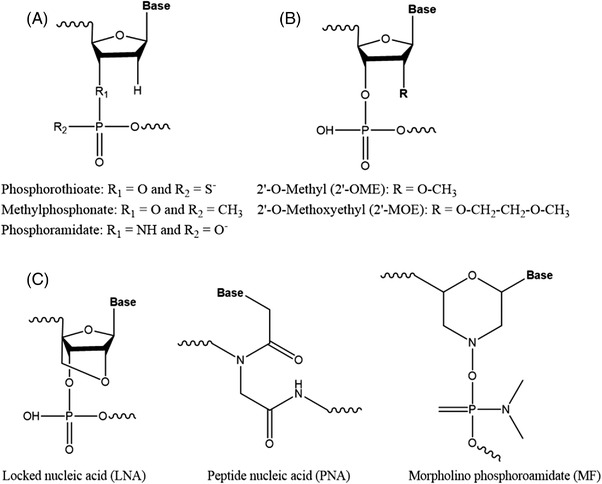
Structure of (A) first, (B) second and (C) third generation of oligonucleotides

**FIGURE 3:**
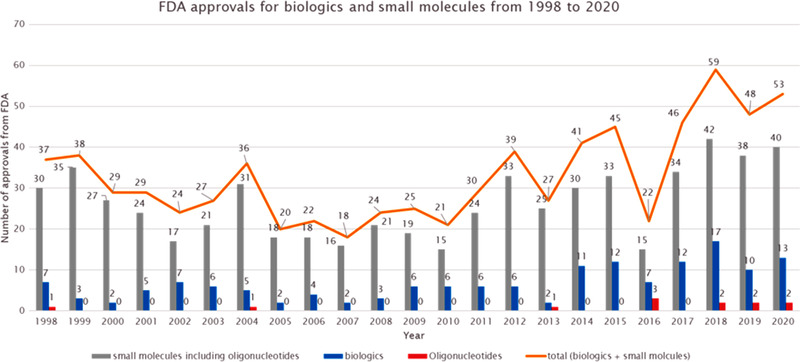
FDA approvals for biologics and small molecules from 1998 to 2020

Oligonucleotides are molecules made up of sugars linked to each other via a phosphate backbone between the carbon 3′ of one sugar to the carbon 5′ of the next one, as shown in Figure 1. Each sugar has a nucleobase on the carbon 1′ which can be thymine, adenine, guanine or cytidine for deoxyribonucleic acid (DNA) and uracil, adenine, guanine or cytidine for ribonucleic acid (RNA). The other difference between DNA and RNA is situated on the carbon 2′ of the sugar, where DNA has H compared to RNA which has an OH group. They can be synthetized with different modifications mainly localised on the phosphate backbone, carbon 2′ of the sugar and/or on the nucleobase.^1^ Those modifications will improve the delivery of the oligonucleotide to the target. Three different generations of oligonucleotides exist depending on the modification applied, as shown in Figure 2. The first generation corresponds to the modification of phosphodiester backbones such as phosphorothioate (PS), methyl phosphonate or phosphoramidate. Then the second generation was developed by modifying the 2′‐hydroxyl group of ribose to increase nuclease resistance while maintaining target binding affinity. This modification includes methoxy and methoxyethyl substituents. Finally, the third generation corresponds to the modification of the furanose ring along with modifications of the phosphate linkage or ribose, as well as of nucleotides. These include locked nucleic acids (LNA), peptide nucleic acids (PNA) and morpholino phosphoroamidates (MF).^1^, ^2^, ^3^, ^4^, ^5^, ^6^ These modifications are used for therapeutic oligonucleotides to target a specific disease that is undruggable by current medicines. The first oligonucleotide therapeutic, Fomivirsen, was approved by the Food and Drug Administration (FDA) in 1998, and since then 11 more oligonucleotides have been approved,^7^ as shown in Figure 3. The oligonucleotides approved by the FDA have different structures such as antisense with PS and/or phosphorodiamidate morpholino backbone, polynucleotide aptamer or mixed single strands of DNA or small interfering RNA (siRNA). They have been categorised as small molecules by the FDA and as new chemical entities by the European Medicines Agency.^8^ Nucleic acids are mainly analysed by ion‐pairing‐reversed‐phase liquid chromatography (IP‐RPLC)^9^ with ultra‐violet (UV) detection and with mass spectrometry (MS), or tandem MS (MS/MS), using electrospray ionisation (ESI) or matrix‐assisted laser desorption/ionisation. These techniques are used to determine their sequence as well as the related impurities, such as *n*‐x or *n*+x (with *n* the full‐length product (FLP) desired and x the number of residue missing or added in the sequence) or other impurities as shown by Pourshahian.^10^ The main fragmentation technique used in MS/MS is collision‐induced dissociation (CID).^11^ Other types of fragmentation techniques have been applied such has as infrared multiphoton dissociation,^12^, ^13^, ^14^ electron photodetachment dissociation,^15^ electron capture dissociation,^16^ electron‐transfer dissociation,^17^, ^18^ electron transfer/collisionally activated dissociation,^18^ negative electron transfer CID,^19^, ^20^ blackbody infrared radiative dissociation ^21^ or electron detachment dissociation.^22^ These techniques will give different ratios of fragment ions, defined by McLuckey nomenclature, and by consequence, they can be used to provide complementary information to obtain a full sequence coverage. Recently, ion mobility spectrometry (IMS) has been used, in conjunction to CID, for the separation of different lengths, identification of w and y fragments^23^ but also to separate different charges,^24^ separate nucleotides, and nucleosides,^25^ separate folded and unfolded oligonucleotides and isobaric ions.^26^ Furthermore, hydrophilic interaction chromatography (HILIC)^27^ and two‐dimensional liquid chromatography (2D‐LC)^28^ have been used as an alternative to IP‐RPLC.^29^

This review covers the different advancements for the analysis and characterisation of oligonucleotides in 2021 by LC‐MS notably by HILIC and 2D‐LC. Also, the different parameters influencing the analysis, such as mobile phase, pH, instrument and column used, the characterisation of methylated nucleobase by IP‐RPLC with MS, and the recent software for oligonucleotides are included.

## 2 PARAMETERS INFLUENCING THE ANALYSIS OF OLIGONUCLEOTIDES BY IP‐RPLC

### 2.1 Influence of the mobile phase

Different parameters can influence the analysis of oligonucleotides when they are analysed by IP‐RPLC.^30^ This has been covered by Guimaraes and Bartlett^31^ where they review the different important factors when nucleic acids are analysed, notably the mobile phase, pH and instrument used. At first, they focused on the use of alkylamine or fluoroalcohol as mobile phase, where hexafluoroisopropanol is used as a weak acid and is more volatile than acetate. This reduces ion suppression and helps to maintain the ion‐pair buffering system. Fluoroalcohol helps in surface desorption by reducing the droplet surface tension. On the other hand, alkylamines help oligonucleotides to reach the surface of the droplet by association with the negatively charged backbone. Finally, they observed that there is no universally best combination of alkylamine and fluoroalcohol for LC‐MS experiments. The disadvantages of alkylamines and fluoroalcohols are that they can cause ion suppression if they are too concentrated and have some solubility problems, which can be solved by using methanol.

**FIGURE 4:**
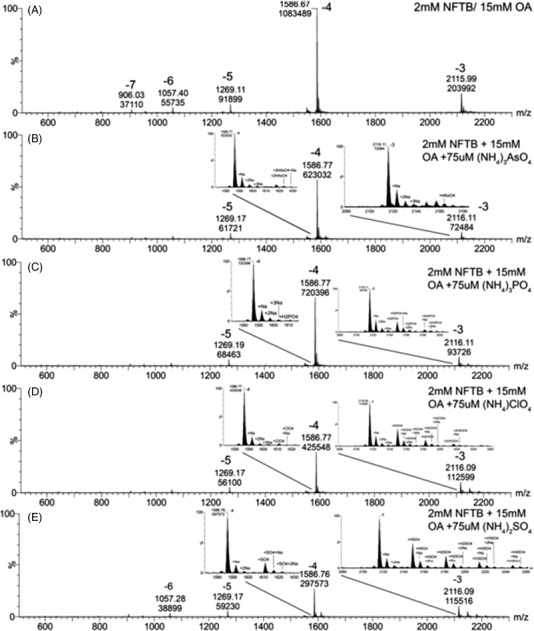
Mass spectra of an oligonucleotide with various anionic salts. (A) Oligonucleotide as control. (B) Oligonucleotide with arsenate adducts. (C) Oligonucleotide with phosphate adducts. (D) Oligonucleotide with perchlorate adducts. (E) Oligonucleotide with sulfate adducts. Y axis, % relative abundance; X axis, m/z. Reprinted with permission from ref 32

In another article, Sutton et al.^32^ also used alkylamine/fluoroalcohol as mobile phase. They demonstrated that using different fluorinated alcohol and alkylamine combinations as mobile phases, such as *nonafluoro‐tert‐butyl* alcohol (NFTB) and octylamine (OA), reduces the charge state envelope for oligonucleotides with ESI. They observed that anions are bound selectively to the low charge states of nucleic acids. To obtain these results, they studied different anions as additives to improve the stability of lower charge states. IMS was used in parallel to determine the effectiveness of promoting secondary structure. Their final mobile phase was 50:50 methanol/ water with 15 mM OA and 25 mM fluorinated alcohol which was applied to two oligonucleotides (20‐mer PS and 32‐mer RNA) by direct infusion and negative ESI. Firstly, they compared NFTB, hexafluoropropan‐2‐ol (HFIP) and ammonium acetate (AA), and observed that AA significantly increases the number of sodium adducts with the presence of higher charge states. When the solvent is changed from pure water to 50/50 water/methanol, the signal intensity increases with a small change of the charge state distribution. HFIP with triethylammonium (TEA) is less acidic and will give higher charge states compared to NFTB with OA. They suggest that the charge state distribution will depend more on the pKa of the mobile phase additive rather than the sample pH. The source temperature also has an impact on the charge state. At lower temperatures, higher charge states are present compared to higher temperatures. They explain this observation due to the desolvation of the solvent. At high temperatures for methanol and OA, they will desolvate quickly and by consequence, the pH will be lower as well as the charge states. When the temperature decreases, more OA will be present in the droplets and so the pH increases, and higher charge state distribution is obtained. When OA is present in the solution, the charge states are lower, independently of the concentration of OA. Then, they evaluated the anionic salt adduction effect by using NFTB/OA, which promotes lower charge states, with the presence of a different ammonium salt. The different salts studied were chloride, bromide, phosphate, iodide, formate, acetate, perchlorate, arsenate and nitrate. None of the salt solutions gives a higher signal intensity compared to when no salt is present. They observed that chloride, bromide, iodide, nitrate, formate and acetate result in the highest signal intensity without salt‐specific anionic adducts present but Na^+^ and K^+^ cationic adducts are still observed. Anionic adduct can be observed for phosphate, perchlorate, arsenate and sulfate, as shown in Figure 4, due to the presence of some extent of hydrogen bonding. These anions can form adducts via hydrogen bonding with the phosphate backbone or the bases. The higher charge states are generally the most abundant. When anions are adducted, they are observed mainly at the lower charge state of the molecule. Finally, the impact of anionic adduction was evaluated using IMS. When adducts are present, no significant increase in drift time is observed, thus, no significant increase in the size. They explain this observation due to the large size of the oligonucleotide compared to the small size of the anionic adduct. The final mobile phase selected for the samples, 50:50 methanol/water with 15 mM OA and 25 mM fluorinated alcohol, should be applied to LC system and compared to the direct infusion and other mobile phases used with LC system.

Roussis, Rodriguez and Rentel^33^ developed another method to increase chromatographic performance and sensitivity. Previously, they observed that the addition of tetraethylammonium bromide (TEAB) eliminates or significantly reduces the formation of K^+^ adduct but gives an ion signal localised on a single charge state. Furthermore, due to the buffering capacity of TEAB, the pH does not need to be further adjusted, similar observations with the use of ammonium bicarbonate (ABC) were made. They could improve the resolution with the presence of small alkyl amines compared to TEAB alone. They observed that the use of propylamine (PA) with a low concentration of ABC produced a higher chromatographic resolution than TEAB and PA. Moreover, the use of triethylammonium acetate (TEAA) with PA gives low resolution with a significant amount of K^+^ adducts. That is why they used 20 mM ABC with 5 mM PA at pH 8.95 as buffer system. By using this IPR, they observed a bimodal charge state distribution where mainly a single charge is obtained at low charges and the remainder distributed over a range of higher charge states. Next, they evaluated which column and mobile phase would be optimal by analysing two oligonucleotides that have phosphodiester backbone, a mix of 2′‐*O*‐methoxyethyl (MOE), and 2′‐deoxy and the presence of cytosine methylated at the 5‐position. They used 20 mM ABC with 5 mM PA at pH 8.95 as mobile phase A and 20% ACN as mobile phase B to compare different columns from Waters, Phenomenex, ACE, Agilent and Thermo. They observed that the Waters Acquity BEH shield RP 18 column provides the highest resolution and, by consequence, they tested it with mobile phase A modified by the addition of hexanoic, octanoic or heptafluorobutyric acids. When no alkyl acid is added, a lower level of separation is obtained. Octanoic acid gives the highest separation for the deoxy series of *n*−1 impurities. A general observation is that the addition of acid allows a better separation for the deoxy series of *n*−1 impurities. They also observed that a long Waters BEH C18 column provided the highest resolution for the *n*‐MOE impurities. They conclude that Waters BEH C18 and Waters BEH Shield RP 18 column provide the highest chromatographic separations for the MOE and deoxy series *n*−1 impurities, respectively. Waters Shield RP18 column also provides adequate resolution of both series, with and without the addition of the alkyl acid and with a shorter run time. This column, without the addition of the acid, can provide adequate separation of the *n*−MOE G and *n*−dG impurities, but cannot separate *n*−dMeC/T and *n*−dA impurities. It is possible to improve the separation with the addition of the alkyl acid only for ndMeC/T impurities but the separation of *n*−dG, *n*−MOE MeC/MeU and *n*−dA is not adequate. They observed that the use of higher charge state ions could generate product ion spectra of considerably higher ion signals and could be used for further MS/MS experiments. They selected 5 mM PA and 0.5 mM octanoic acid as a suitable IP reagent with or without the addition of alkyl acid. Furthermore, multiple reaction monitoring (MRM) can be used on a triple quadrupole mass spectrometer to determine co‐eluting impurities and to detect isobaric impurities notably for *n*−dA isomer impurity. By using this method, they could resolve *n*–1 impurities.

As observed, alkylamine/fluoroalcohol are still the most used mobile phase to improve the sensitivity and the chromatographic performance in IP‐RPLC. Different combinations of alkylamine and fluoroalcohol for LC‐MS experiments are possible and there is no universal one. In general, TEA is used with HFIP but other types are used as shown here such as NFTB with OA to impact the charge envelope with the addition of anions or the use of ABC with PA and with or without alkyl acid such as octanoic acid.

### 2.2 Influence of the pH, instruments and materials used

**FIGURE 5:**
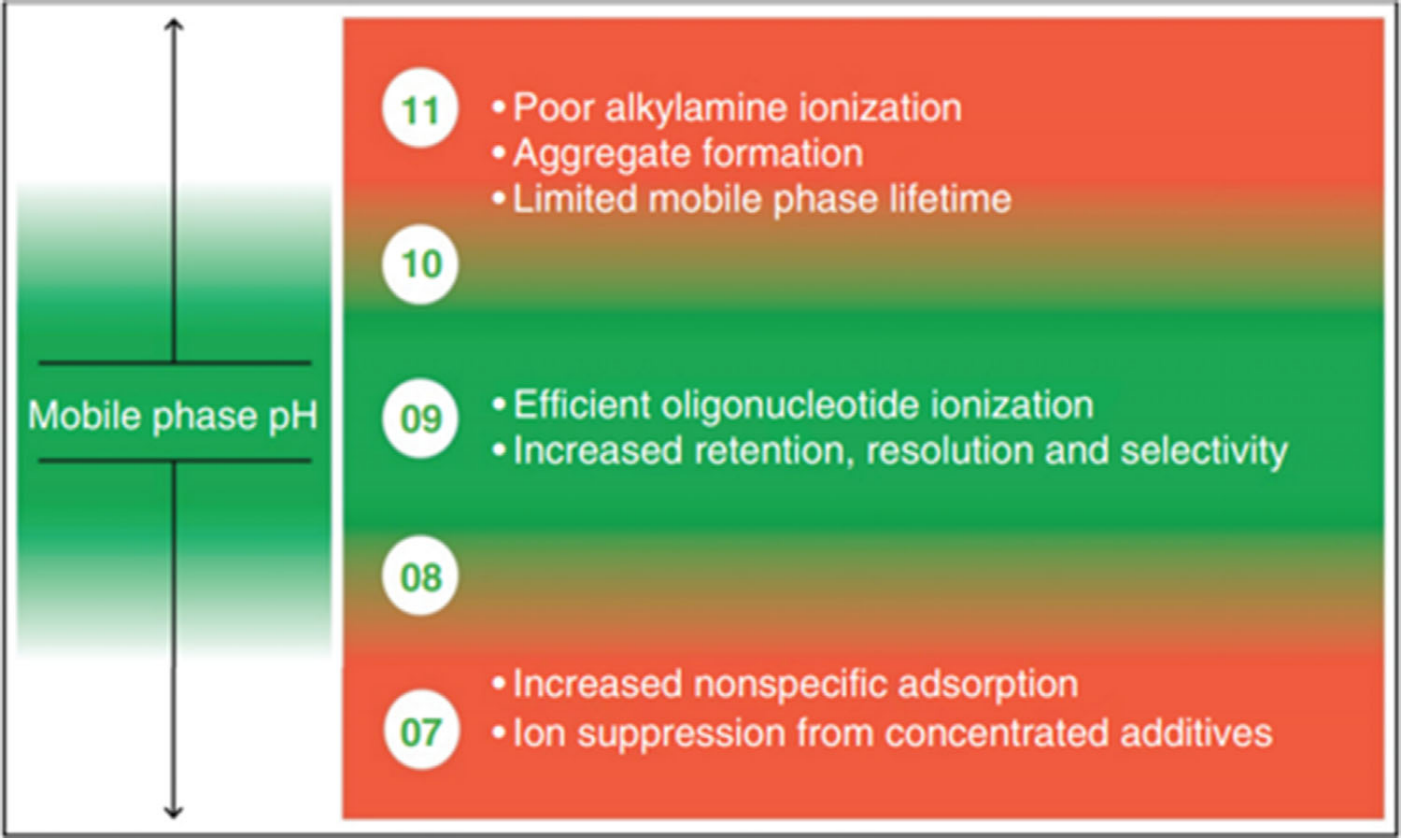
General relationship between mobile phase pH and method performance. Reprinted with permission from ref 31

As mentioned previously, Guimaraes and Bartlett^31^ also evaluated the influence of the pH and instrument used when oligonucleotides are analysed by IP‐RPLC. They suggest avoiding contact of the oligonucleotide with nucleases on the skin of the scientist during analysis, as this can cause degradation of the compound. As a consequence, nucleic acids should be manipulated with gloves and in a disinfected/clean area. Another problem is the high surface activities of the materials used, notably glass; hence, only plastics should be used to avoid non‐specific adsorption losses of oligonucleotides. Nucleic acids will also be adsorbed on metal‐oxide surfaces present in the LC, such as the injector, in‐line filters, tubing and columns, which can become positively charged. As a consequence, these elements need to be passivated to avoid adsorption losses. Different techniques exist, such as conditioning the system with a series of injections of the analyte which will saturate the different sites or more often by injecting a surrogate oligonucleotide to occupy the different non‐specific binding sites, but which is temporary. Another method is to add different additives in the mobile phase that helps to minimise the sample adsorption, such as citrates, phosphates, ethylenediaminetetraacetic acid, acetylacetone or medronic acid, but may have a negative impact on MS sensitivity. Moreover, phosphoric or citric acid solutions wash can be used to passivate the entire LC‐MS system. Finally, the use of hybrid surface technology, such as columns, is more and more used which does not need any conditioning.^34^ Furthermore, they assessed the impact of the pH on oligonucleotides. At high pH, the alkylamine becomes neutral and starts to form micelles in the mobile phase which is favoured by an elevation of the column temperature. This will be translated by a change in the retention time. To avoid the formation of micelles, the pH needs to be below 7.5 and the mobile phase should be made fresh daily. If no water is used, the mobile phase can be stable for a week. One of the advantages of working at high pH is that the charges are reduced for the metal surfaces in the chromatographic system which will decrease but will not eliminate non‐specific adsorption. On the other hand, working at low pH values will not have significant consequences but there may be some chromatographic alterations, notably the increase of the surface charging of metal surfaces. By consequence, the capacity of these metal surfaces to non‐specifically adsorb oligonucleotides will increase. In other words, it will be more complicated to passivate the system and maintain it during the analysis. Finally, the authors proposed a general relationship between pH and mobile phase performance as shown in Figure 5. They also conclude with some recommendations, such as using alkylamine at a concentration between 5 and 15 mM, to have the possibility to adjust the pH easily. It will also provide the optimal response for ESI. This concentration is important to have a good mass spectral response and an easy titration of the pH to avoid micelles and ion suppression. On the other hand, fluoroalcohols should be between 30 and 40 mM to avoid ion suppression. They recommend the use of hybridized metal surfaces in chromatographic systems and columns to reduce non‐specific adsorption.

### 2.3 Impact of the choice of the column

As seen previously, the column and the ion‐pair reagent have an impact on the separation of oligonucleotides. Chen, Liu and Gong^35^ have evaluated five C18 columns with the same dimension but packed with core‐shell silica, polymer, porous silica and hybrid particles, respectively, as shown in Table 1, and six ion‐pairing reagents (IPRs) (*N*,*N*‐dimethylcyclohexylamine [DMCHA], *N*,*N*‐dimethylbutylamine [DMBA], TEA, tripropylamine [TPA], *N*‐diisopropylethylamine [DIPEA] and hexylamine [HA] with HFIP). They compared the retention behaviour and chromatographic resolution for different oligonucleotides sequences under different mobile phase conditions in negative ESI mode with a quadrupole mass spectrometer. They used identical LC conditions for all experiments but with different IPR. They observed that the polymer column produced poor chromatography peak shape for every experiment. Conversely, silica‐based porous particles retained homogeneous and heterogenous oligonucleotides strongly independently of the IPR used compared to C18 columns packed with core‐shell or hybrid fully porous particles. Furthermore, all C18 columns showed an order of elution of A, then T and then C for HA but a different one when any other IP‐HFIP is used such as C, then A and then T. When HA is used, they observed that the retention on the column was dependent on the stationary phase and oligonucleotide sequence. Moreover, the separation of heterogeneous nucleic acids is highly dependent on the mobile phase composition, the column stationary phase and the oligonucleotide sequence, where IPR impacts the separation independently of C18 columns. On the other hand, for homogeneous oligonucleotide, the IPR, oligonucleotide sequence and length could have an impact on the separation for the C18 columns. Also, large particles will reduce the retention of oligonucleotides independently of the IPR used for homogeneous, heterogeneous and modified oligonucleotides. The highest separation was with HA and the lowest separation when TEA was used for homogeneous and heterogeneous. There is no significant difference in retention for the homogeneous and heterogeneous sequence when DIPEA, DMCHA, DMBA or TEA is used with BEH C18, Oligo BEH C18 or Oligo MS C18 columns. But when TPA is used, an order of retention can be determined such as Oligo BEH C18 > Oligo MS C18 > BEH C18 only for the same homogeneous nucleic acid. For heterogeneous oligonucleotides, oligo BEH C18 and Xbridge BEH C18 columns produced better separation than the other columns when the buffer was DIPEA and HFIP. But Oligo BEH C18 column delivered the worst separation for the same analysis of oligonucleotides with TEA. Furthermore, when TPA was the IPR, Super C18 failed to separate heterogeneous nucleic acid, but all other C18 columns can achieve baseline separation. When two heterogeneous oligonucleotides cannot be separated by HA‐HFIP, all other IPR can with a baseline separation and vice versa. They observed that the retention for different types of columns, for modified oligonucleotides (LNA, methylation, phosphorylation, PS and fluorescent), is similar to the heterogeneous sequences. Regardless of the types of C18 columns, HA‐HFIP gives the strongest retention. Super C18 column gives the longest retention time with the worst separation for phosphorylated with DIPEA‐HFIP and TPA‐HFIP. As well, when TPA was used, Super C18 and Oligo MS C18 columns were the worst for the separation of PS. They concluded that Super C18 would be the best for the separation of methylated with HA or Oligo MS C18 with TPA. On the other hand, Oligo BEH C18 should be used with TEA‐HFIP to separate phosphorylated oligonucleotides. Moreover, when LNA is analysed, HA could be the best, independently of the column. TEA would be the best for the detection of modified nucleic acid when HFIP is used in MS, independently of the column. The choice of IPR and the column is important when modified oligonucleotides are analysed compared to non‐modified sequences. The type of column had no significant impact on the MS detection or does not affect the ionization efficiency of nucleic acids for heterogeneous and homogeneous sequences. Only the IPR influences the MS signals for heterogeneous nucleic acids. HA with HFIP gives the highest ionisation efficiency and would be the best for homogeneous and heterogeneous analysis. They advised that TPA and DMCHA would be a good IPR for the separation of heterogeneous nucleic acids, regardless of the type of C18 column used for the analyses. The type of IPR used in the mobile phase and the nucleic acid sequence property, such as length and base composition, as well as modification have a significant impact on MS signals of synthetic and chemically modified oligonucleotides. As observed here, HA is the IPR that produced the longest retention time for all oligonucleotide independently of the C18 particles. Furthermore, when the LC conditions are identical, C18 columns performed similarly for the separation of nucleic acids but depend on the type of IPR and oligonucleotide sequence. The MS signal can be slightly impacted by the type of particles used but a significant impact is observed depending on the IPR and sequence of synthetic and chemically modified oligonucleotide used. Finally, depending on the type of oligonucleotide analysed, it is important to select the correct IPR and column.

**TABLE 1:** The different C18 chromatography columns evaluated in the study of Chen, Liu and Gong. Reprinted with permission from Ref. [35]

Column	Dimension	Particle type
ACQUITY UPLC BEH C18 (BEH C18)	2.1 × 50 mm, 1.7 μm, 130 Å	Fully porous BEH C18 hybrid particles
XBridge Oligonucleotide BEH C18 (XBridge BEH C18)	2.1 × 50 mm, 2.5 μm, 130 Å	Fully porous BEH C18 hybrid particles
ACQUITY UPLC Oligonucleotide BEH C18 (Oligo BEH C18)	2.1 × 50 mm, 1.7 μm, 130 Å	Fully porous BEH C18 hybrid particles
Clarity Oligo‐MS C18 (Oligo MS C18)	2.1 × 50 mm, 1.7 μm, 100 Å	Core‐shell silica‐based C18
ACE Excel Super C18 (Super C18)	2.1 × 50 mm, 1.7 μm, 90 Å	Fully porous silica‐based C18
PRP‐C18	2.1 × 50 mm, 5 μm, 100 Å	PS‐DVB functionalised with C18

## 3 CHARACTERISATION OF METHYLATED NUCLEOBASES BY IP‐RPLC

As explained previously, oligonucleotides can be modified at different sites of the molecule which will improve the delivery of the oligonucleotide to the target. Recently, more focus has been done on the third generation, which has higher stability in biological fluids, is resistant to degradation by nucleases and peptidases and have a strong hybridisation affinity with the messenger RNA. By consequence, different new methods exist to characterise and identify this type of modification particularly for methylation situated on the nucleobase.^1^ Boulias and Greer^36^ proposed an optimised protocol for the detection and quantification of methylated nucleobases including *N*6‐methyladenine (6mA), *N*4‐methylcytosine (4mC) and C5‐methylcytosine (5mC) in genomic DNA. They used LC coupled to a triple quadrupole mass spectrometer using positive ESI to obtain an accurate quantification of DNA methylated thanks to the high sensitivity of the instrument and the use of calibration standards. Their first step to optimise the method was to analyse individually the different modified and unmodified nucleosides by UHPLC‐MS/MS to determine their retention time. Then, the detection and quantification are performed in dynamic multiple reaction monitoring mode, by monitoring the *m/z* transitions from precursor to product ion for dA, 6mA, 4mC, 5mC and dC. More precisely, the quantification is carried out by quantifying the ratio of 6mA/dA, 5mC/dC or 4mC/dC. In other words, the method is based on the retention time and *m/z* of the desired precursor and product ions.

Another recent method has been developed by Yan et al.^37^ to characterise modified transfer RNA (tRNA). This method is mainly focused on the use of site‐specific RNase H digestion with LC−MS/MS for tRNA. After digestion, the products were ionised by negative ESI with the use of Q‐TOF‐MS/MS. They obtained single nucleotides and nucleobases by fragmenting the different oligonucleotides by CID. They could identify the different fragments by using the accurate mass of nucleotides and nucleobases with or without modifications and compared them to a customised database. They evaluated where the modification was present, in the base, ribose or phosphate groups, by comparing the *m/z* of the monomethylated base to the deprotonated free base. Finally, they used the retention time of different standards to compare it to the sample to identify the position of the modification. This method can be used if only one modified base is present in the portion of the digestion to identify its modification and its position in the sequence. If two modifications are present in the portion, another mode needs to be used in the software RNA ModMapper (RAMM). By using a site‐specific RNase H cleavage, a better sequence coverage is obtained compared to conventional RNase digestion. This difference is due to the length of the product obtained where site‐specific RNase H cleavage gives fragments of length 8 to 14 mer compared to conventional RNase digestion which gives too short fragments and so cannot be placed onto the sequence. Another disadvantage of this site‐specific method is that it consumes a lot of samples and takes a long time which could be improved by parallel analysis.

**FIGURE 6:**
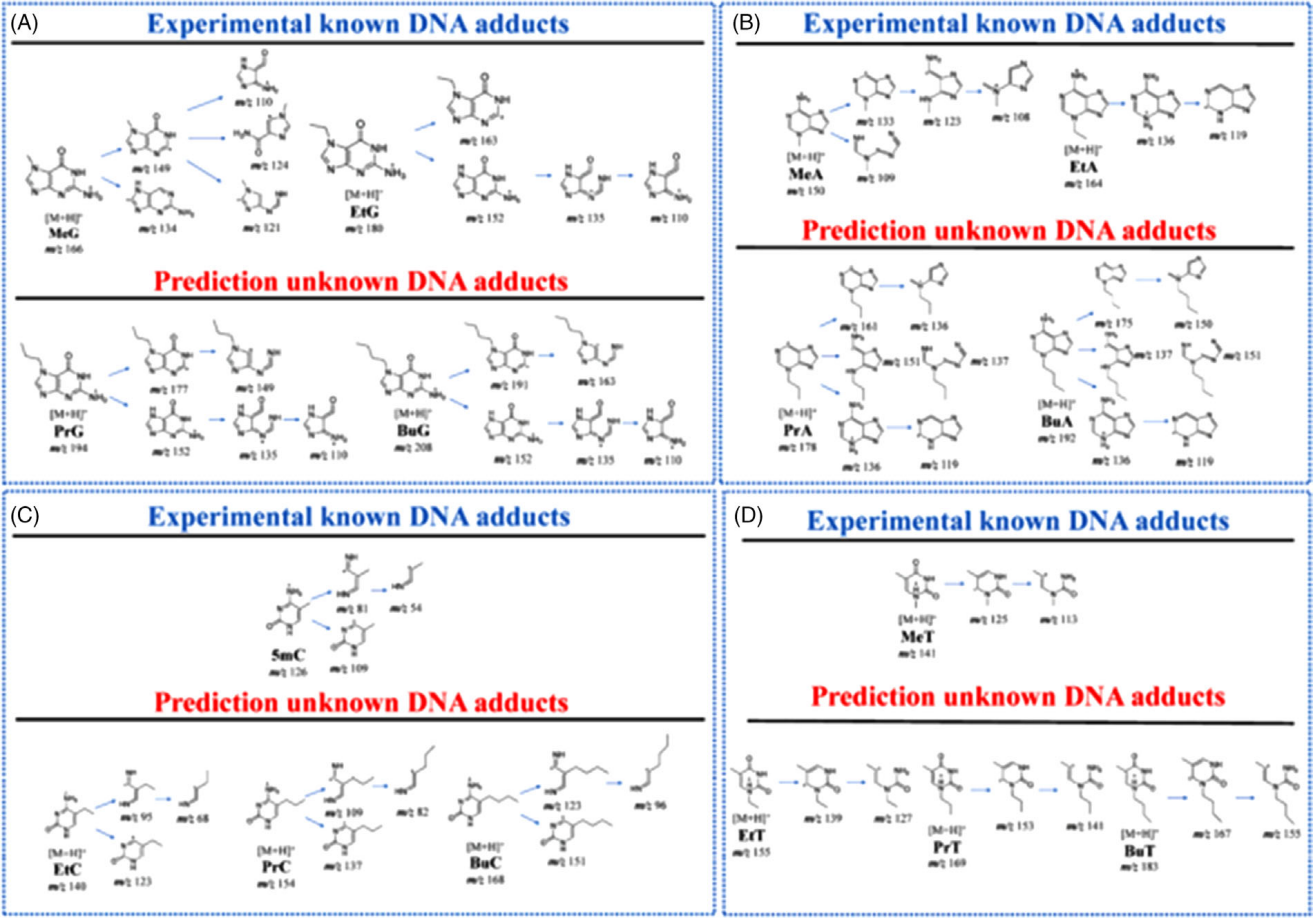
Prediction of unknown DNA adducts from experimental known DNA adducts (A: guanine, B: adenine, C: cytosine and D: thymine). Reprinted with permission from ref 38

Another approach has been proposed by Tang et al.^38^ to characterise 7‐propyl guanine (7PrG) and 7‐butyl guanine (7BuG) adducts using MRM signals of DNA with high‐resolution MS data and synthetic standards. Their method is based on the prediction of MRM which includes 36 possible precursor ions and characteristic product ion transitions of DNA adducts. This method has been applied to a sample of human cell and rat tissues after nitrosamine and sulfonate exposure. They could observe different alkyl methanesulfonates such as methyl methanesulfonate, ethyl methanesulfonate, *N*‐propyl methanesulfonate and *N*‐butyl methanesulfonate, which leads to the formation of 7‐methyl guanine in addition to their specific alkylation DNA adducts. They developed a prediction to identify unknown DNA adducts after exposure to sulfonates by MRM profiling MS strategy. To do that, they predicted the MS fragmentation of unknown DNA adducts based on the fragmentation of known DNA adducts by MRM in positive mode for methyl guanine, ethyl guanine, methyl adenine, ethyl adenine, methyl cytosine and methyl thymine, as shown in Figure 6. They give an example for propyl and butyl guanine fragmentation where the prediction involves heteroatom dealkylation, deamination and ring opening of the purine based on the fragments. Consequently, by knowing which part of the molecule is lost, they could focus their analysis on a specific fragmentation pathway and use MRM. For example, they target a specific fragment transition, such as for propyl guanine, they were looking for the transition *m/z* 194 to 152. They carried out this experimentation for 7PrG, 1‐propyl guanine and 6‐propyl guanine. Thanks to these results, they could determine where the modification was situated for unknown DNA samples. They predicted the MRM list of adducts for the four different DNA bases and obtained 36 predicted species. They could confirm the presence of 7PrG and 7BuG in human cells by comparing the retention times of the six standards and using the prediction‐driven MRM profiling MS strategy. The limitation of this technique is the localisation of this modification in the sequence, the separation and resolution of the other bases and the use of standards to confirm the reaction sites of the unknown DNA adducts.

Finally, Sun et al.^39^ quantitated the level of DNA cytidine methylation (5mC) and hydroxymethylation (5hmC) by direct injection and compared it to LC‐MS. To do that, after digestion and desalting, they analysed the sample using Advion TriVersa NanoMate, to avoid the carryover of samples by direct infusion and an Orbitrap (Thermo Scientific). The analysis was less than 1 min and had a detection limit of approximately 0.3 ng/mL compared to using nano LC where the lowest LOD was 0.04 ng/mL. In the first instance, they optimised the sample buffer composition, source fragmentation energy, the radio frequency of the instrument ion funnel and in‐source fragmentation to improve sensitivity and reduce the formation of nucleoside byproducts such as dimers. Finally, they could quantify 81 samples of modified DNA in 1.5 h. This method is fast, accurate, precise, cost‐effective and bypasses common LC‐MS issues such as sample carryover, lack of sensitivity due to poor binding of nucleosides to the column and imperfect resolution of the peaks by chromatography. Moreover, it also overcomes biases in differential chromatographic retention and issues of sample degradation in the autosampler. They tested different organic solvents and acid concentrations to obtain a better MS signal. They observed that 70% of organic solvent, such as ACN or methanol with 0.1% of formic acid (FA), gives an intense signal by direct infusion. However, without the use of FA, a better signal is obtained for 70 to 80% of ACN. They quantified 5mC and 5hmC by generating a calibration curve to obtain a ratio for the modified nucleosides compared to the unmodified by direct infusion and LC. They observed that by direct infusion, a more similar ratio is obtained compared to the analysis performed by LC. Furthermore, a simpler signal extraction in direct infusion acquisition is obtained. Finally, direct infusion reduces the bias in quantifying nucleosides by providing a similar limit of quantification for 5mC and 5hmC compared to LC. This method can be limited to only pure sample and does not show where exactly the modification is situated, but just the presence of it. Furthermore, other types of modifications need to be evaluated. As observed here, the most recent analysis has been focussing on the characterisation and quantification of methylated nucleobase, which corresponds to the third generation of oligonucleotides. The disadvantage of the different methods is the lack of exact localisation of the modification in the sequence. As a consequence, a new type of characterisation needs to be developed to answer this problem.

## 4 ADVANCE IN THE CHARACTERISATION OF OLIGONUCLEOTIDES BY HILIC

**FIGURE 7:**
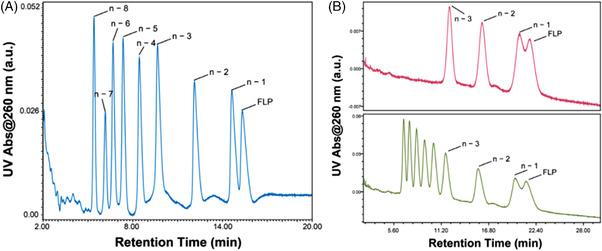
(A) HILIC‐UV chromatograms showing the separation of the mixture of synthetic 3′ (n − x) truncated sequences of a DNA oligonucleotide. (B) HILIC‐UV chromatograms showing the separation of the 4‐oligo (upper panel) and 9‐oligo (lower panel) mixture of synthetic 3′ (n − x) truncated sequences of a PS oligonucleotide. Reprinted with permission from ref 27

HILIC,^40^ compared to IP‐RPLC, does not use IPRs which could cause some potential ionisation suppression and contamination. That is why it can be a good alternative to analyse oligonucleotides. Huang et al.^27^ have developed a new method using HILIC‐MS/MS with a Waters BEH amide column with high‐resolution MS with a quadrupole orbitrap and heated ESI in negative mode with high‐energy C‐trap dissociation (HCD). The use of targeted selected ion monitoring (tSIM) or parallel reaction monitoring (PRM) allows them to improve the sensitivity. They evaluated the separation, mass determination, sequence characterisation, impurity profiling and quantitation for unmodified and fully PS oligonucleotides and siRNAs. Their method uses 70% of acetonitrile (ACN) buffered with AA or ammonium formate (AF) as mobile phase A and 30% of ACN buffered with AA or AF as mobile phase B, where AA and AF are two commonly used additives in the mobile phases to obtain good chromatographic peak shapes and MS response with different elution strengths. This method is used to analyse siRNA, DNA, RNA and related impurities. They defined a retention time order for oligonucleotides as duplexed RNA > RNA oligonucleotide > DNA oligonucleotide > phosphate backbone fully PS modified DNA oligonucleotide. When PS is analysed, it diminishes the retention due to higher hydrophobicity compared to the phosphate backbone. Firstly, AF and AA were compared at a concentration at 25 mM and obtained comparable peak capacities with a slightly smaller retention factor (*k*) for AF. Then, they compared AF at 25 mM, which gives the lowest MS response, to AA at different concentrations, where 15 mM of AA gives the highest MS response. They also observed that at low salt concentration, the HILIC retention time decreases with more charge state distribution at low *m/z* values. Then, they tested different pH values at fixed AA concentrations. At high pH (with ammonium hydroxide), the HILIC retention time is lower compared to low pH (with acetic acid) which has peak tailing without affecting the LC peak resolution. Furthermore, the column temperature has been optimised. They observed that at elevated temperatures, such as 80°C, the column retention increases as well as the peak capacity compared to low temperature (30°C) for single stranded. When a duplex is analysed, at high temperature (70°C), it is completely or partially melted and so peak broadening is observed with elevated retention time. Below the melting point, the duplex remains intact and by consequence, a low temperature of column, such as 30°C, needs to be chosen to preserve the structure of the nucleic acid. They chose 15 mM of AA (pH 9.0) with a column at 30°C to have a good chromatographic performance and reasonable MS signal intensities. Finally, a low abundance of metal adduct is seen due to the AA which aids the removal of alkali metal adducts with replacement by volatile ammonium ions. They advised that when the Waters BEH amide column is used, the method should be optimised by screening salt concentrations and pH. They applied this method to evaluate the separation of impurities to the FLP. For a DNA of 25 mer, all 3′ (*n*–x) impurities were separated from the FLP. However, when the sequence is fully PS‐modified, they could not fully resolve the 3′ (*n*–1) impurity from the FLP, while the other 3′ (*n*–x) sequences eluted closer together. Only 3′ (*n*–3) and 3′ (*n*–2) could be resolved, as shown in Figure 7. They explain this observation due to the presence of diastereoisomers. Furthermore, HILIC separation is only based on hydrophilicity which is not variable between oligonucleotides due to the presence of phosphate groups. In contrast, IP‐RPLC is based on hydrophobic interactions between nucleic acid bases and reversed‐phase column chemistries, but also from the different hydrophobicities of the individual base which contributes to the separation. As a result, HILIC is less robust than IP‐RPLC due to fewer interactions. Impurity profiling was possible by extracted ion chromatograms using the *m/z* value of the most abundant precursor ions of each (*n*–x) sequence from either 3′ or 5′ terminus to calculate signal intensities. To characterise oligonucleotides, the authors isolated the most abundant precursors ions and applied a panel of % normalized collisional energy (%NCE) values ranging from 13 to 23%. The precursor, with higher charge states, requires higher NCE for good sequence coverage compared to the precursor with low charge states. They defined an optimal NCE at 15% for a 90% sequence coverage of [M–5H]^5−^ by using the software Biopharma FinderTM 4.0 (Thermo Fisher Scientific). But, for [M–6H]^6−^ they did not obtain a good sequence coverage. The HCD has been optimised to unbiased sequence characterisation of DNA and RNA by MS/MS, which improves their sequence coverage. Finally, they carried out quantitative analysis by selecting PSO_2_^−^ with PRM. PSO_2_^−^ is more prevalent at higher %NCE compared to low %NCE which gives comprehensive sequence annotation. They evaluated the column sensitivity by performing tSIM or PRM, where tSIM gives higher *S/N* ratios and sensitivity (injection of 13 ng equivalent to 2.0 pmol) compared to PRM.

The analysis of oligonucleotides by HILIC‐MS avoids some constraints of IP‐RPLC and it can be fast, sensitive and repeatable. HILIC can also provide separation, mass determination, sequence characterization, impurity profiling and quantitation. This method has some limitations, notably for the separation of PS backbone impurities, but could be optimised. Furthermore, it would be interesting to evaluate this method for other types of modified nucleic acids notably for oligonucleotides with a modification on the 2′ sugar or at the nucleobase.

## 5 ADVANCES IN THE CHARACTERISATION OF OLIGONUCLEOTIDES BY 2D‐LC

Recently, 2D‐LC^41^ has been applied to oligonucleotides. Li and Lämmerhofer developed a method to evaluate the impurities present from the antisense strand of Patisiran by using three different 2D‐LC platforms.^42^ They used a quinine (QN) carbamate‐based weak anion‐exchange (AX) stationary phase (QN‐AX) and a classical C18 RP stationary phase in IP mode with tripropylammonium acetate (TPAA), respectively, in the first dimension (^1^D) to provide the selectivity of impurities formed during the synthesis. Firstly, they evaluated the Chiralpack QN‐AX column with a mixed pH/triethylammonium phosphate (TEAP) buffer gradient in ^1^D. They observed that a start at low pH is necessary for the resolution of impurity peaks. The optimisation of the flow rate of the gradient allows them to have a better peak capacity. They observed that a higher flow rate will increase the peak capacity with more resolved peaks. For the use of QN‐AX, they conclude to use a mixed pH/TEAP gradient with a constant organic modifier of 20% ACN. They also assessed a classical IP‐RPLC with TPAA as the IPR using an Acquity UPLC Oligonucleotide BEH C18 column in ^1^D multiple‐heart‐cutting (MHC) and comprehensive 2D‐LC approach. They used TPAA for stronger retention and better resolution. They included TPAA only in mobile phase A due to a too long analysis when this one was included in both mobile phases. With a gradient of 20 min, they could separate 27 peaks with a good peak capacity. In contrast, when TEAA was used, a gradient of 40 min was necessary and lower resolution was observed. Both ^1^D methods are MS‐incompatible and by consequence, RP‐column is used in the second dimension (^2^D) for desalting and removal of non‐volatile phosphate buffer components and IPRs. This allowed the analysis using MS by negative ESI of resolved impurity peaks. Without IPRs in the 2D‐RPLC eluent, no further selectivity between different oligonucleotides is achieved and, hence, all nucleic acids from different cuts elute at the same retention time. This assures identical ionization conditions in the ESI source which makes the oligonucleotide signals more comparable with loss of selectivity. They used MHC on impurities and high‐resolution sampling on the main peak, using the two different ^1^D methods with the determination of the impurities by MS. When the purity of the main peak was determined by comprehensive 2D‐LC approach with the QN‐AX column in the 1D, they could detect *n*−1 and *n*−2 shortmers at the beginning of the main peak and no impurities on the middle part. However, they could not obtain any fractions with pure FLP using the C18 column. Moreover, they could identify 18 different impurities when the QN‐AX column was used in heart cutting compared to 17 when the C18 was used. They obtained a higher limit of detection (LOD) for QN‐AX compared to the C18 but when the entire peak is used for integration, a similar LOD is obtained. This shows the advantages of using a QN‐AX column compared to a C18. Finally, they coupled QN‐AX LC in ^1^D with the IP‐RPLC with TPAA in the ^2^D with UV detection for online analysis and impurity profiling. They used comprehensive 2D‐LC approach to analyse the main peak and MHC for the impurity peaks. This allows the separation of additional impurities which co‐eluted in the 1D. They needed to adjust the method only for the ^2^D part of the IP‐RP. The downside with this method is that two runs are necessary as the fraction storage in the loops for ^1^D is limited and the ^2^D chromatographic run is not fast enough. They observed that more peaks could be separated in the ^2^D by MHC IP‐RPLC in UV. This method is not MS‐compatible and by consequence, the impurities cannot be characterised by MS. QN‐AX and IP‐RP are complementary techniques to separate impurities, but some optimisations are still necessary notably in the ion source parameters to improve the sensitivity. Furthermore, they need to evaluate this method on other types of drugs and oligonucleotides with different modifications.

## 6 RECENT DEVELOPMENT OF SOFTWARE FOR OLIGONUCLEOTIDES

To help the process of different complex data obtained after analysing oligonucleotides by tandem MS, some software exists such as OMA & OPA,^43^ Mongo Oligo,^44^ RAMM^45^ or RoboOligo.^46^ A recent program has been developed by Agten et al.,^47^ which is an online tool to predict the aggregated isotope distribution for a sequence of DNA or RNA up to a mass of 25 kDa. This software will predict the first 20 isotope peaks for DNA and RNA molecules. The monoisotopic mass of the molecule needs to be entered and the representation of the predicted isotope distributions is shown. This software is limited only to the monoisotopic mass of neutral molecule and it is not possible to have multiply charged states. Furthermore, it is only possible to use it for DNA and RNA with a phosphodiester backbone and unmodified with a sequence from 5 to 92 mer for DNA and 5 to 90 for RNA. This is translated by a mass of 1463.2424 to 30290.8424 Da and 1543.2170 to 31072.2797 Da, respectively. This software can be used to detect and deconvolute nucleic acid isotope patterns in a mass spectrum for which the elemental composition is unknown. Other recent software is available commercially, such as BioPharmaFinder or PMI‐Byos Oligo workflow (Protein Metrics Inc.), which gives more options notably for sequence determination by MS/MS.

## 7 CONCLUSIONS

The use of therapeutic oligonucleotides as medicines is increasing though few have been approved by the FDA and many are currently in development for different diseases such as Duchenne muscular dystrophy, spinal muscular atrophy or for cancers.^7^, ^48^, ^49^, ^50^, ^51^ More generally, nucleic acids are used in the area of metabolic disorders, oncology, neurology, ophthalmology cardiovascular, muscular diseases, gastrointestinal, infectious diseases, genitourinary, dermatology, haematology, respiratory, hormonal disorders and immunology.^6^ That is why it is important to have a better characterisation of their sequence and impurities. This has been possible by optimising and choosing the optimal mobile phase and column in IP‐RPLC but also with the recent methods developed for HILIC and 2D‐LC, which are more newly applied techniques. Furthermore, more focus has been carried out on the characterisation of the third generation of oligonucleotides and most particularly on methylated nucleobases. Finally, there is still a lack of the development of the software to help to process the complex data obtained after analysing oligonucleotides by tandem MS, particularly for unknown impurities. That is why *de novo* sequencing needs to be developed for new software.

## CONFLICT OF INTEREST

The authors have declared no conflict of interest.

## AUTHORS CONTRIBUTION

Fabien Hannauer: writing–original draft, review and editing (lead); G. John Langley: review (equal); Eugen Stulz: review (equal); Andrew D. Ray: review (equal); Stephen W. Holman: review (equal); Rachelle Black: review (equal).

## Data Availability

Data sharing not applicable ‐ no new data generated, or the article describes entirely theoretical research.
